# Three new species of the genus *Stephos* Scott, 1892 (Crustacea, Copepoda, Calanoida, Stephidae) from Jeju Island, Korea

**DOI:** 10.3897/zookeys.944.49361

**Published:** 2020-06-30

**Authors:** Seong Yong Moon, Ho Young Soh, Dae Hyun Cho

**Affiliations:** 1 South Sea Fisheries Research Institute, National Institute of Fisheries Science, Yeosu 59780, South Korea National Fisheries Institute of Fisheries Science Yeosu South Korea; 2 Department of Environmental Oceanography, Chonnam National University, Yeosu 596166, South Korea Chonnam National University Yeosu South Korea; 3 Department of Oceanography, Chonnam National University, Gwangju 61186, South Korea Chonnam National University Gwangju South Korea

**Keywords:** *
Stephos
*, new species, benthopelagic, near bottom, Jeju Island

## Abstract

During general field surveys carried out recently to collect benthopelagic copepods from near the substrate of the shallow waters off Jeju Island, Korea, a few specimens of three new species of *Stephos* Scott, 1892, were collected. The new species are placed in the genus *Stephos* because of the following combination of features: absence of seta on the basal exite of maxillule, and male right leg 5 ending in an unarmed claw-like and/or mitten-like segment. *Stephos jejuensis***sp. nov**. can be distinguished from its congeners by body length 0.92 mm, left side of the female genital double-somite with protruding lobes, antennule that extends beyond the distal area of the genital double-somite, and the male leg 5 terminal complex. *Stephos concavus***sp. nov**. can be distinguished from its congeners by the genital double-somite with protruding lobes on both sides, and the presence of larger spinules on the distomedial margin of leg 5. *Stephos fortipes***sp. nov.** can be distinguished from its congeners by its longer body length, 1.12 mm long in the female, antennules that extend to the end of the genital double-somite, and the presence of a covered row of minute spinules on the ventral surface of the genital operculum in the female. Until now, 35 species of stephids were known worldwide.

## Introduction

Benthopelagic copepods are of low abundance and high diversity in the benthic boundary layer (Bradford-Grieve 2004). However, they are difficult to sample on the continental slope or ocean-basin environments, which has contributed to the slow accumulation of knowledge about the benthopelagic fauna (Bradford-Grieve 2004). The benthopelagic calanoid family Stephidae Sars, 1902, consists of four valid genera by: *Stephos* Scott, 1892; *Parastephos* Sars, 1902; *Miostephos* Bowman, 1976 and *Parastephos* Sars, 1902; *Speleohvarella* Kršinić, 2005. Their species are generally smaller in body size, and include hyperbenthic forms living in anchialine and marine coastal habitats (Boxshall and Halsey 2004; Jaume et al. 2008; Kršinić 2012, 2015; Moon et al. 2015; Suárez-Morales et al. 2017). The genus *Stephos* is the most diverse, comprising 32 species (Bradford-Grieve 1999; Boxshall and Halsey 2004; Kršinić 2015; Moon et al. 2015; Suárez-Morales et al. 2017). Up to now, there have been a total of eleven species from the Australia-Western Pacific region, as follows: *S. pentacanthos* Chen & Zhang, 1965; *S. morii* Greenwood, 1978; *S. tropicus* Mori, 1942; *S. tsuyazakiensis* Tanaka, 1967, *S. pacificus* Ohtsuka & Hiromi, 1987; *S. angulatus* Bradford-Grieve, 1999; *S. robustus* Ohtsuka & Hiromi, 1987; *S. kurilensis* Kos, 1972; *S. hastatus* Bradford-Grieve, 1999; *S. geojinensis* Moon, Youn & Venmathi Maran, 2015; and *S. projectus* Moon, Youn & Venmathi Maran, 2015. Species of *Stephos* show many similarities to species of its confamilial genera but differs as follows: the male right fifth leg is 4-segmented (vs. 5-segmented in *Parastephos* and 3-segmented in *Miostephos* and *Speleohvarella*); and the male right fifth leg ends in an unarmed claw-like and/or mitten-like segment (vs. a claw being armed with spines along the concave margin in *Parastephos* and reduced in *Miostephos* and *Speleohvarella*) (Boxshall and Halsey 2004). The zoogeographical distribution of species of *Stephos* was established by Suárez-Morales et al. (2017).

During a survey of the copepod fauna of the southern coasts of Jeju Island, the largest island in Korea, a few specimens of stephids were collected from near-bottom shallow waters by vertical tows of 0.1-mm mesh conical nets at high tide in dusk hours. One of these samples contained representatives of several *Stephos* not known to the benthopelagic environment. This paper reports on three undescribed species of the genus *Stephos* that are herein described in full and compared with their known congeners around the world.

## Materials and methods

**Figure 1.**
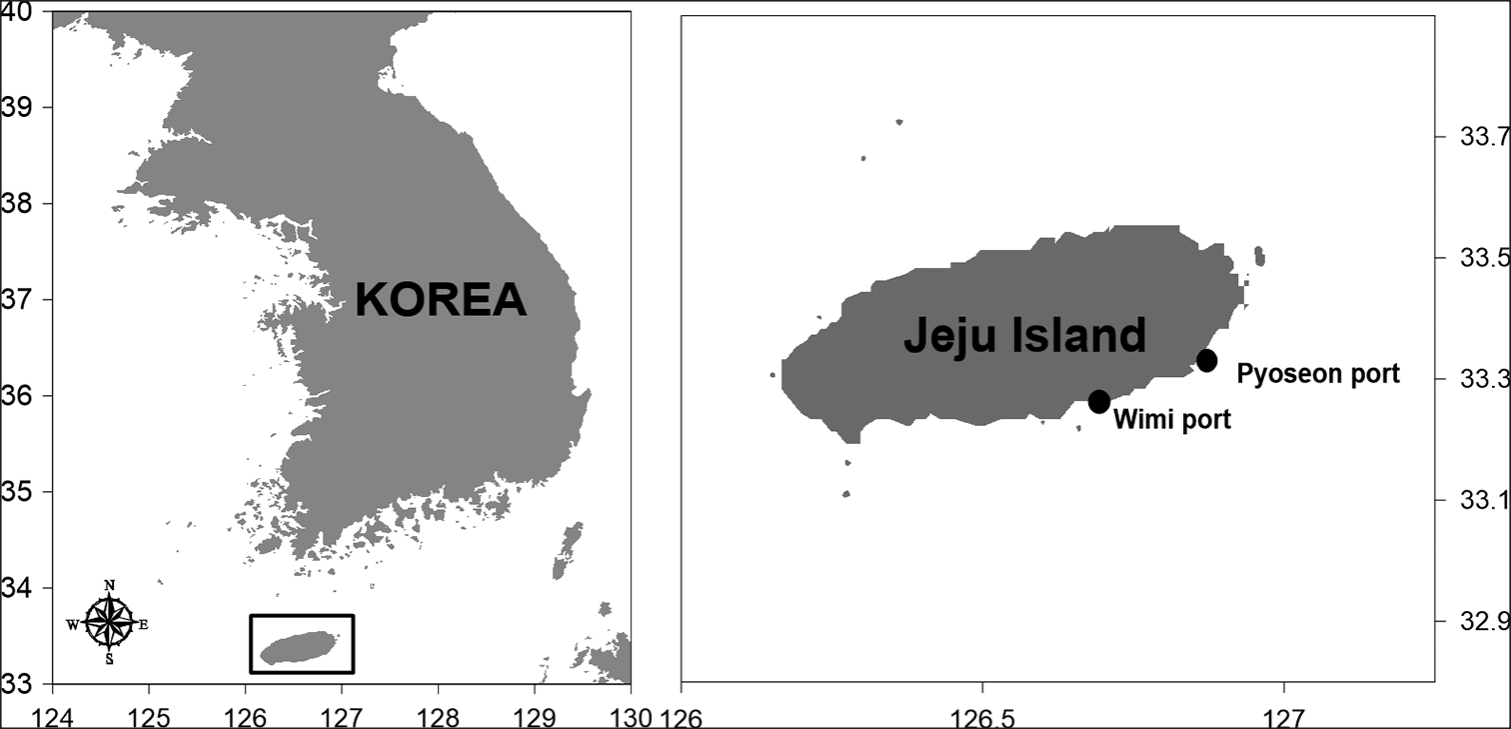
Map showing the sampling location (black circles) in Jeju Island, Korea.

Copepods were collected from the shallow waters of Jeju Island, Korea by vertical tows (0.1-mm mesh conical nets) at high tide in dusk hours (Fig. 1). For morphological examination, samples were fixed in a 5% natural formalin-seawater solution and cleared in 70% lactic acid for an hour before dissection in a drop of lactophenol on a wooden slide under the dissection microscope (Humes and Gooding 1964). Dissected body parts and appendages were examined under a compound microscope with magnification up to X1,000. Measurements were made with a stage micrometer from the head to the tip of the caudal ramous, excluding the caudal setae. Drawings were made with the aid of a drawing tube equipped on the microscope. The morphological terminology follows Huys and Boxshall (1991) and Ferrari and Ivanenko (2008). An abbreviation used in the text and figures is ae, for aesthetasc. Specimens are deposited at the National Institute of Biological Resources (**NIBR**), Incheon, Korea.

## Taxonomy

### Order Calanoida Sars, 1901

Family Stephidae Sars, 1902

Genus *Stephos* Scott, 1892

#### Stephos jejuensis sp. nov.

Taxon classificationAnimaliaCalanoidaStephidae

9CAAB50A-1D81-593B-AFFC-6528673FE433

http://zoobank.org/94A56606-DD69-43B6-9EB0-F6ED232AF163

**Figure 2.**
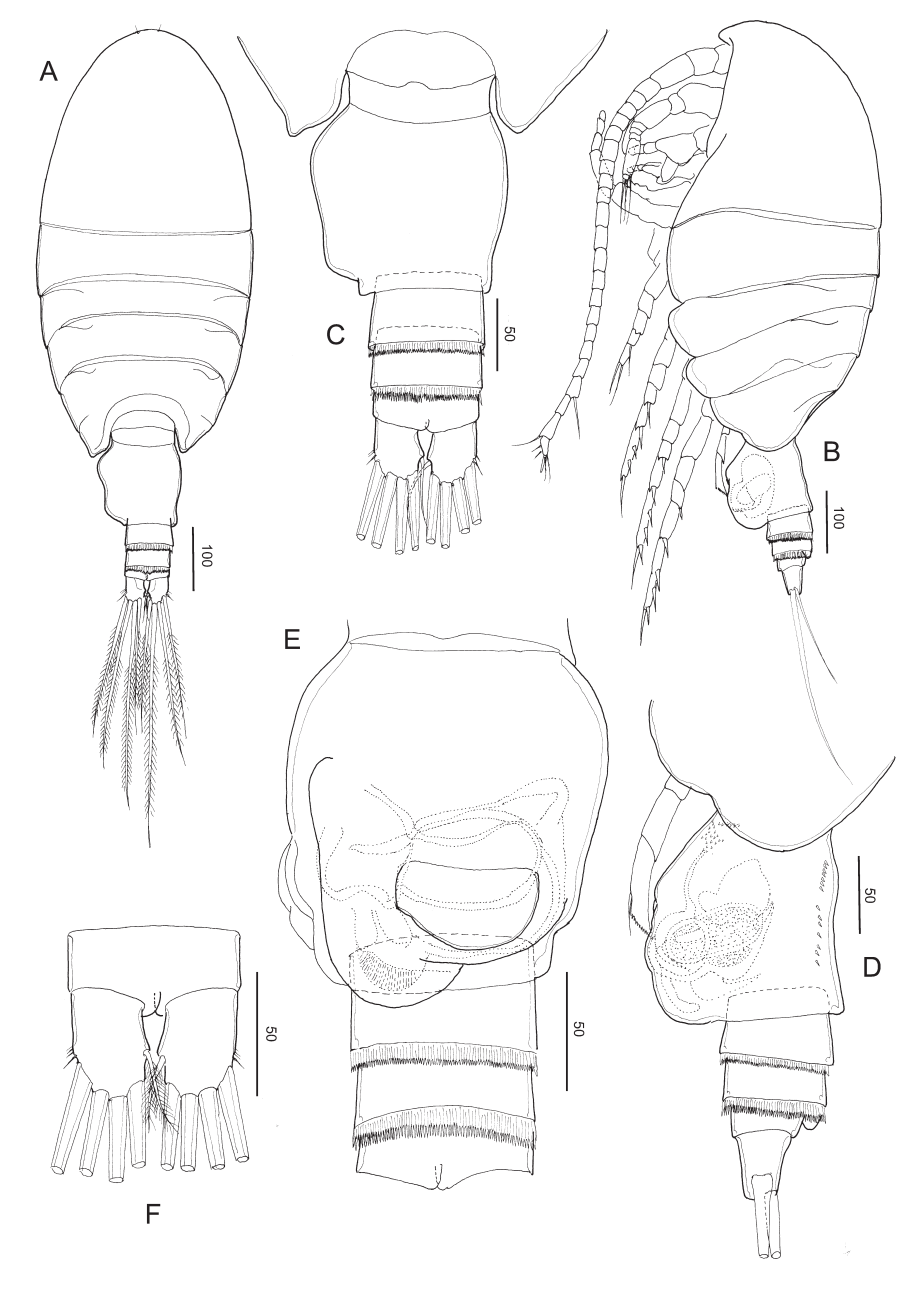
*Stephos jejuensis* sp. nov. Female paratype **A** habitus, dorsal view **B** habitus, lateral view **C** urosome, dorsal view **D** urosome, lateral view **E** urosome, ventral view **F** anal somite and caudal rami. Scale bars in µm.

**Figure 3.**
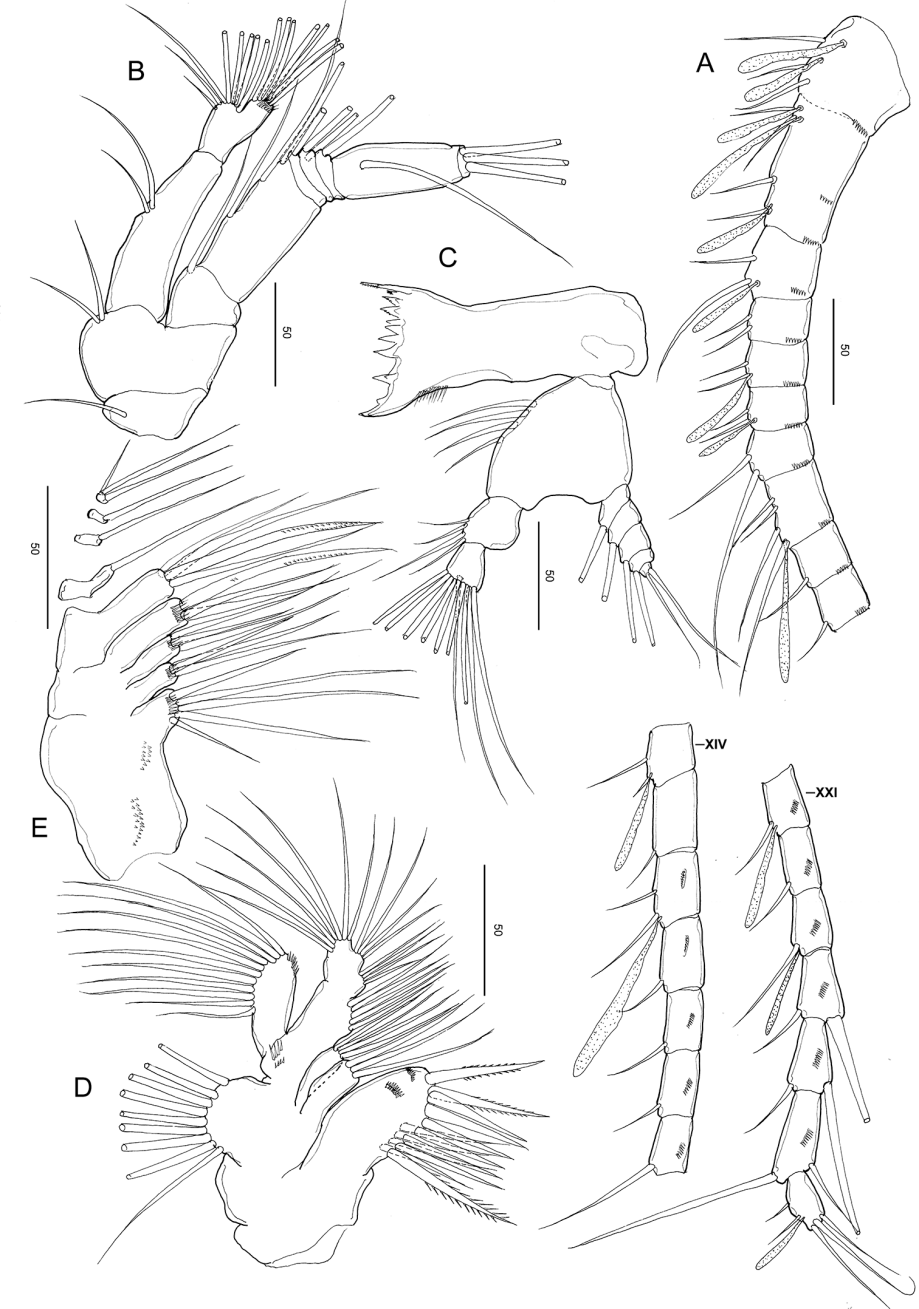
*Stephos jejuensis* sp. nov. Female paratype **A** antennule **B** antenna **C** mandible **D** maxillule **E** maxilla. Scale bars in µm.

**Figure 4.**
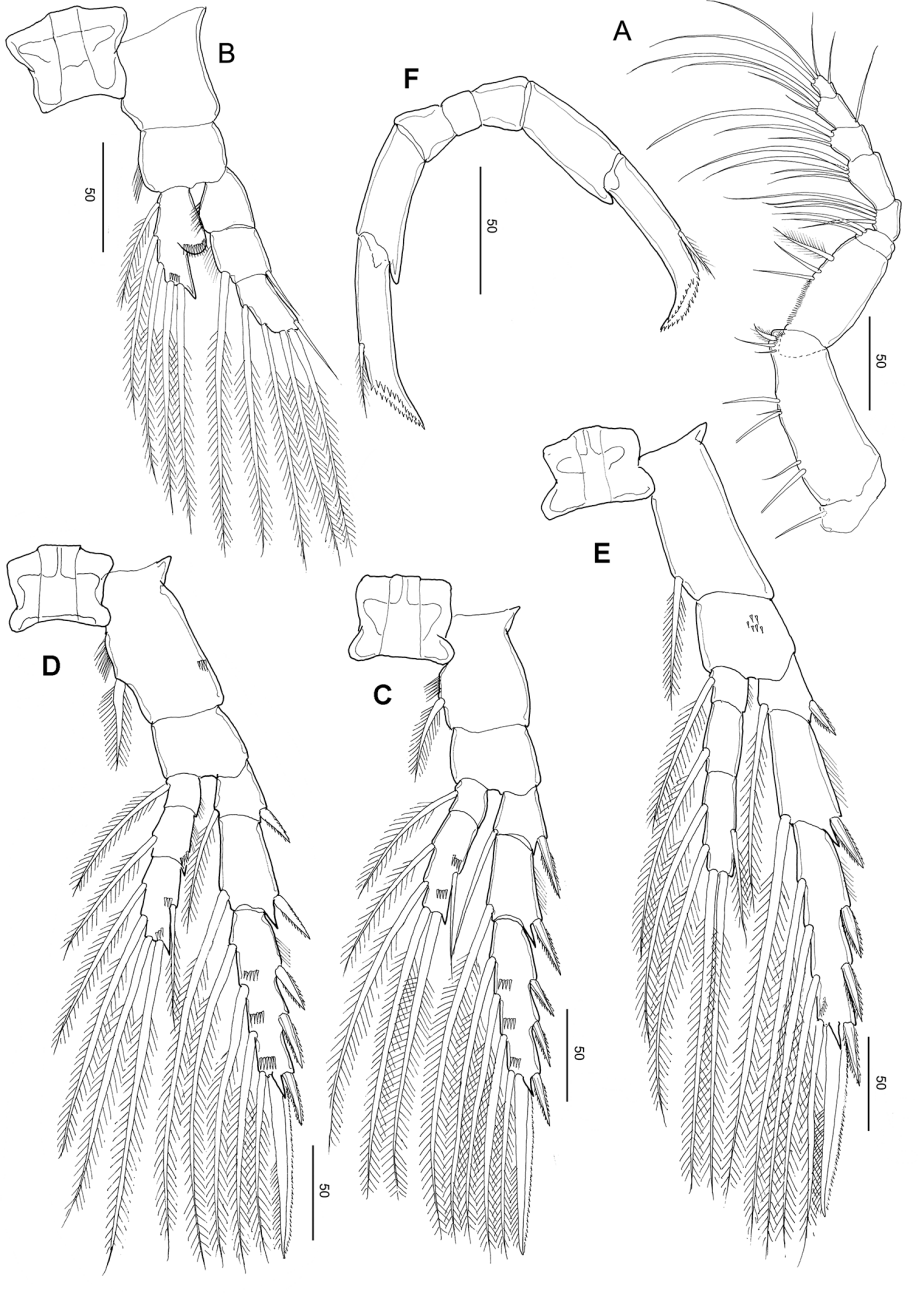
*Stephos jejuensis* sp. nov. Female paratype **A** maxilliped **B** leg 1 **C** leg 2 **D** leg 3 **E** Leg 4 **F** Leg 5. Scale bars in µm.

**Figure 5.**
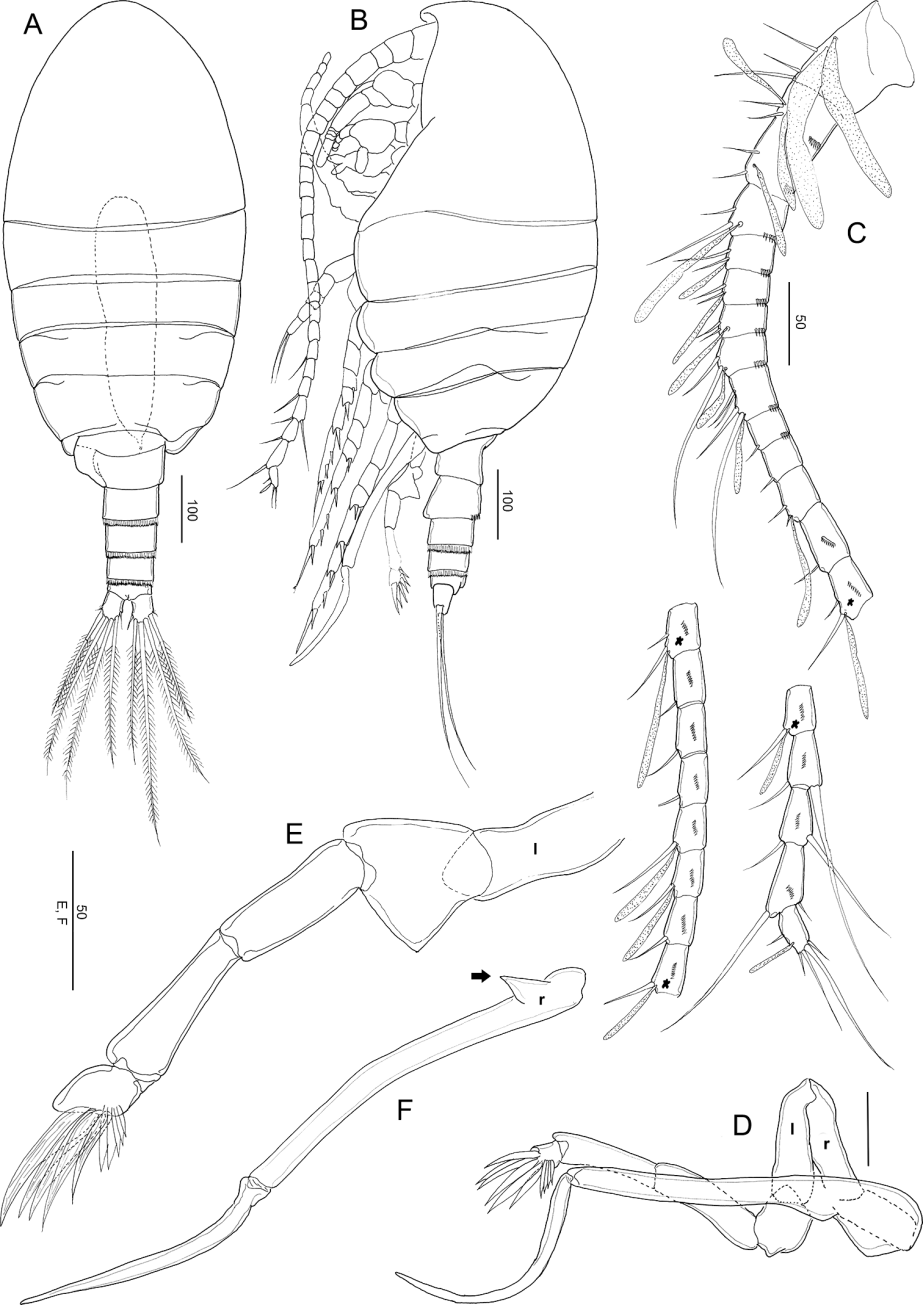
*Stephos jejuensis* sp. nov. Male paratype **A** habitus, dorsal view **B** habitus, lateral view **C** antennule **D–F** leg 5. Scale bars in µm.

Figures 2 , 3 , 4 , 5

##### Material examined.

***Holotype*** ♀ (NIBRIV0000840220), allotype ♂ (NIBRIV0000840219) undissected in 70% ethanol, 11 November 2012. Dissected ***paratypes*** ♀ (NIBRIV0000840221), ♂ (NIBRIV0000840222) mounted on two glass slides, 11 November 2011. All specimens collected by D. H. Cho.

##### Type locality.

Near the bottom (ca. 5 m depth), Pyoseon port, Jeju Island (33°19'32"N, 126°50'42"E), Korea.

##### Etymology.

The specific name of the new species *jejuensis* refers to the type locality.

##### Description of female.

***Body*** (Fig. 2A, B) robust, length 0.92 mm (mean 0.91 ± 0.03, *N* = 3). Prosome 5-segmented; cephalosome and first pedigerous somites completely separated; fourth and fifth pedigerous somites incompletely fused (Fig. 2A, B), posterior corners of fifth pedigerous somite slightly asymmetric. Rostrum represented by a rounded knob. Prosome-urosome ratio 2.61:1. Urosome 4-segmented, comprising genital double-somite, two free abdominal somites, and anal somite; length ratio of genital double-somite, first free abdominal somite, second free abdominal somite, and anal somite as 48.2: 14.2: 12.6:11.7:13.4 = 100. Genital double-somite (Fig. 2C, E) asymmetric, with protruding lobe on the anterior to posterior of the left side and a projecting lobe to distal margin, with minute spinules patched in lateral view (Fig. 2D); on the right anterior side is a swollen, common operculum bumpy-shaped ventromedially and with ear lobe on the ventrolateral margin. First and second abdominal somites with transverse hyaline frill dorsally and ventrally. Anal somite short. Caudal rami (Fig. 2F), with six setae, symmetric, 1.45 times longer than wide (44 × 31 μm); caudal setae II–VII present (seta I lacking); seta II spiniform, seta III ca. half the length of seta V, seta V longer (right longer than left) than seta IV, both plumose; dorsal seta VII short, plumose.

***Antennule*** (Fig. 3A) symmetric, extending beyond the distal area of genital double-somite; 24-segmented, apparently ancestral. Segments I–II, III–IV, X–XI, and XXVII–XXVIII are fused. Segmentation and setation pattern as follows (ancestral segment number-setae+aesthetasc): I–II-3+2ae, III–IV-4+3ae, V-2+ae, VI-2, VII-2+ae, VIII2+ae, IX-2, X–XI-4+ae, XII-1, XIII-1, XIV-2+ae, XV-1, XVI-2+ae, XVII-1, XVIII-1, XIX-1, XX-1, XXI-1+ae, XXII-1, XXIII-1+ae, XXIV-1+1, XXV-1+1, XXVI-1+1, XXVII–XXVIII-5+ae. Ancestral segments I to XIV and XVI to XXV with a row of spinules on the posterior surface.

***Antenna*** (Fig. 3B) biramous; coxa and basis separate, coxa with one and basis with two setae; endopod 2-segmented, proximal segment with two setae, compound distal segment bilobed with eight and seven plumose setae subterminally and terminally, respectively, outer margin ornamented with small serrated process subdistally on the medial margin; tiny spinule adjacent to serrated process; exopod 7-segmented, with intersegmental articulation between segments 2 and 3 not completely expressed, with setal formula of 1, 3, 1, 1, 1, 1, 3.

***Mandible*** (Fig. 3C): well-developed coxal gnathobase, with a straight row of moderately incised teeth. Mandibular palp biramous; basis with four setae on the inner margin. Exopod 5-segmented, with setal formula of 1, 1, 1, 1, 2; endopod 2-segmented, proximal with four setae and distal segments with ten setae.

***Maxillule*** (Fig. 3D): praecoxal arthrite bearing nine stout marginal spines and four elements on posterior surface, rows of tiny spinules on the posterior surface. Coxal epipodite with nine setae; coxal endite with three stiff setae. Basis with cluster of denticles on the anterior surface; proximal basal endite with four setae; distal basal endite indistinct, with five setae; no trace of basal exite. Exopod with eleven marginal setae and a row of setules along the distal portion of the medial margin. Endopod not articulated to basis, indistinctly 3-segmented, setal formula 4, 4, 7.

***Maxilla*** (Fig. 3E): apparently 6-segmented, comprising coalesced praecoxa and coxa, allobasis and 3-segmented endopod. Armature of praecoxal and coxal endites 5,3,3,3. Basal endite with four setae, one stouter than the rest; endopodal endite with one seta on tip. Free endopod setal formula 1, 1, 3 respectively. Integument of praecoxa ornamented with patch of spinules on the posterior margin. Praecoxal and coxal endites with cluster of long spinules subdistally on the lateral surface; distal coxal endite with additional row of spinules proximally on the medial surface.

***Maxilliped*** (Fig. 4A): syncoxa robust, with setal formula 1, 2, 2, 3 and oblique row of tiny spinules on the posterior distal part; basis with three setae and a row of setules on the mediolateral margin; endopod 6-segmented, with setal formula 2, 4, 4, 3, 3+1, 4.

***Legs 1–4*** (Fig. 4B–E) progressively larger toward the posterior, each comprising coxa, basis, and 3-segmented exopod; endopod of leg 1 (Fig. 4B) 1-segmented, that of leg 2 (Fig. 4C) 2-segmented; endopods of P3 (Fig. 4D) and leg 4 (Fig. 4E) 3-segmented. Armature formula of legs 1–4 as follows (Roman numerals indicate spines, Arabic numeral indicates setae):

**Table:** 

Legs	Coxa	Basis	Exopod	Endopod
Leg 1	0-0	0-1	0-0; I-1; I,1,3	0,2,3
Leg 2	0-1	0-0	I-1; I-1; III,I,4	0-1; 1,2,2
Legs 3 and 4	0-1	0-0	I-1; I-1; III,I,4	0-1; 1,2,2

Leg 1 (Fig. 4B) biramous, with long curved inner setae on the basis, and endopod with lobe on the outer margin, bearing a minute spinous process and a row of minute spinules on the anterior surface.

Leg 2 (Fig. 4C) biramous, endopod 2-segmented; coxa and basis unarmed; second endopodal segments with a row of spinules on the medial and distal edges, with a pointed process on the distolateral corner; exopod 3-segmented, with a row of spinules on the medio to distal margins of the distal exopodal segment.

Legs 3 (Fig. 4D) and 4 (Fig. 4E) biramous, with 3-segmented rami: coxa and basis unarmed; second and distal endopodal segments with a row of spinules on the distal edges, with a pointed process on each of the distolateral corners; exopod with a row of spinules on the medial to distal margins of the distal exopodal segment.

Leg 5 (Fig. 4F) symmetric, uniramous, 3-segmented with a proximal segment fused to intercoxal sclerite; basis separated, 2.27 times longer than wide (41 × 18 μm), widening distally with minute spinules on the anterior corner and an acute inner process, and unarmed. Distal segment with a transverse row of spinules across near the middle part and an outer seta medially.

##### Description of male.

***Body*** (Fig. 5A, B) robust, length 0.93 mm. Prosome 5-segmented; cephalosome and first pedigerous somites completely separated; fourth and fifth pedigerous somites incompletely fused (Fig. 5A). Rostrum same as in female. Prosome-urosome ratio 2.18:1. Urosome 5-segmented, comprising genital somite, three free abdominal somites, and anal somite; length ratio of genital somite, first to fourth free abdominal somites, and anal somite as 27.5: 19.2: 16.3: 14.5: 10.9: 11.5 = 100. Genital somite with asymmetric and protruding lobe on the left side. First to third abdominal somites with transverse hyaline frill dorsally and ventrally. Anal somite shortest. Caudal rami similar to those of the female.

***Antennule*** (Fig. 5C) symmetric, extending beyond the distal area of the genital double-somite; 24-segmented, apparently ancestral; segments I–II, III–IV, X–XI, and XXVII–XXVIII are fused. Segmentation and setation pattern as follows (ancestral segment number-setae+aesthetasc): I–II-3+2ea, III–IV-4+3ae, V-2+ae, VI-2, VII-2+ae, VIII2+ae, IX-2, X–XI-4+ae, XII-1, XIII-1, XIV-2+ae, XV-1, XVI-2+ae, XVII-1, XVIII-1, XIX-1, XX-1, XXI-1+ae, XXII-1, XXIII-1+ae, XXIV-1+1, XXV-1+1, XXVI-1+1, XXVII–XXVIII-5+ae. Ancestral segments I–XIV and XVI–XXV with row of spinules on the posterior surface.

***Antenna***, mandible, maxillule, maxilla, maxilliped and legs 1–4 similar to those of the female.

***Leg 5*** (Fig. 5D–F), strongly asymmetric, slender on both sides, developed as a grasping organ on the left. Right leg 4-segmented; coxa and basis are short, unarmed, but thickened proximally; terminal segment comprising a single longer process (see arrowed in Fig. 5F), outwardly directed, curved medially, and acute at its tip. Left leg 5-segmented (see Fig. 5E); proximal segment ca. as long as right proximal segment; second segment with rounded outgrowth on medial margin; third segment elongated, unarmed; fourth segment narrow, shorter than third segment; terminal segment complex, with 5 terminal (long) and 5 subterminal (short) lamella spines.

##### Variations.

Within this new species, there was a minor variation in the number of spinules on the genital double-somite and on the surfaces of legs 1–4 in both sexes.

##### Remarks.

The genital double-somite in most species of *Stephos* has been found to be symmetric and/or slightly asymmetric in shape. The feature of an asymmetric genital somite in *S. jejuensis* sp. nov. is shared with five of its congeners, *S. lamellatus* Sars, 1902; *S. tsuyazakiensis* Tanaka, 1966; *S. exumensis* Fosshagen, 1970; *S. kurilensis* Kos, 1972; and *S. robustus* Ohtsuka & Hiromi, 1987. Of these, *S. jejuensis* has a projecting lobe on the distal margin in the lateral side of the genital double-somite; however, the other five species do not have this feature. *Stephos jejuensis* has been group IV.

In addition, *S. jejuensis* expresses by two diagnostic features: the fifth pedigerous somite is slightly asymmetric; and a projecting lobe in the lateral side of the genital double-somite. These features are shared by only one other species: *S. jejuensis* can be distinguished from *S. maculosus* (Bradford-Grieve 1999) by the following features in the female: the body length is 0.92 mm (vs. 0.62 mm in *S. maculosus*); dorsally the left side of the genital double-somite has anterior and posterior protruding lobes (vs. without protruding lobe in *S. maculosus*); the antennule extends beyond the distal area of the genital double-somite (vs. not beyond the distal area in *S. maculosus*); and the distal segment is less than four times longer than the second segment of leg 5 (vs. more than four times in *S. maculosus*). In the male: the body length is 0.93 mm (vs. 0.54 mm in *S. maculosus*); the antennule extends beyond the distal area of the genital double-somite (vs. beyond the anterior margin of the caudal rami in *S. maculosus*); on the leg 5 fourth segment of the male is narrow (vs. with an finger-like lobe on the medial expansion in *S. maculosus*); and the leg 5 terminal segment complex consists of five terminal (long) and five subterminal (short) lamella spines (vs. not complex, only with three lamella spines in in *S. maculosus*).

#### Stephos concavus sp. nov.

Taxon classificationAnimaliaCalanoidaStephidae

069DFB13-0DFC-515E-8972-A1767130E483

http://zoobank.org/812ECEFE-53ED-4675-90C9-D631F3D40F0C

Figures 6 , 7 , 8

##### Material examined.

***Holotype*** ♀ (NIBRIV0000293109) dissected on two glass slides collected by D. H. Cho, 9 May 2012.

##### Type locality.

Near the bottom (ca. 4 m depth), Wimi port, Jeju Island (approximately 33°16'13"N, 126°39'43"E), Korea.

**Figure 6.**
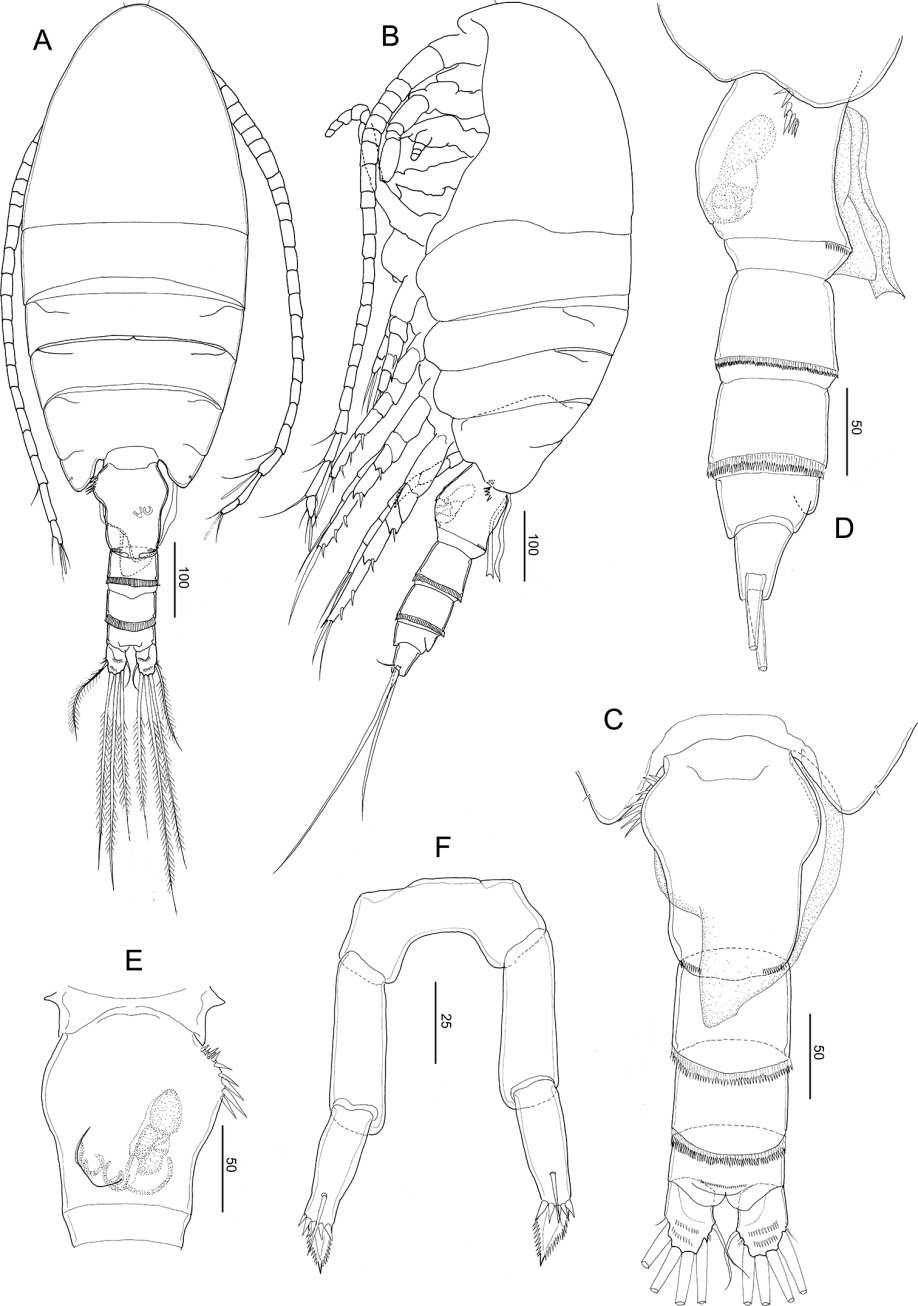
*Stephos concavus* sp. nov. Female holotype **A** habitus, dorsal view **B** habitus, lateral view **C** urosome, dorsal view **D** urosome, lateral view **E** genital double-somite, ventral **F** leg 5. Scale bars in µm.

##### Description of female.

***Body*** (Fig. 6A, B) robust, length 0.93 mm. Prosome 5-segmented; cephalosome and first pedigerous somites completely separated; fourth and fifth pedigerous somites incompletely fused (Fig. 6A), posterior corners of prosome slightly asymmetric. Rostrum represented by a rounded knob. Prosome-urosome ratio 2.25:1. Urosome 4-segmented, comprising genital double-somite, two free abdominal somites, and anal somite; length ratio of genital double-somite, first free abdominal somite, second free abdominal somite, and anal somite as 43.0: 18.9:18.1:9.4:10.7 = 100. Genital double-somite (Fig. 6C–E) slightly asymmetric, with protruding lobe on the anterior to medial part of both sides and with a row of spinules in lateral view (Fig. 6C, D); common operculum located ventromedially slightly round (Fig. 6E) and with spermatophore and coupler in dorsal view (Fig. 6C). First and second abdominal somites (Fig. 6C), with transverse hyaline frill dorsally and ventrally. Anal somite shortest. Caudal rami with six setae, symmetric, 1.75 times longer than wide (49 × 28 μm); caudal setae II–VII present (seta I lacking); seta II spiniform, seta III ca. half the length of seta V, seta V longer (right longer than left) than seta IV, both plumose; dorsal seta VII short, plumose.

**Figure 7.**
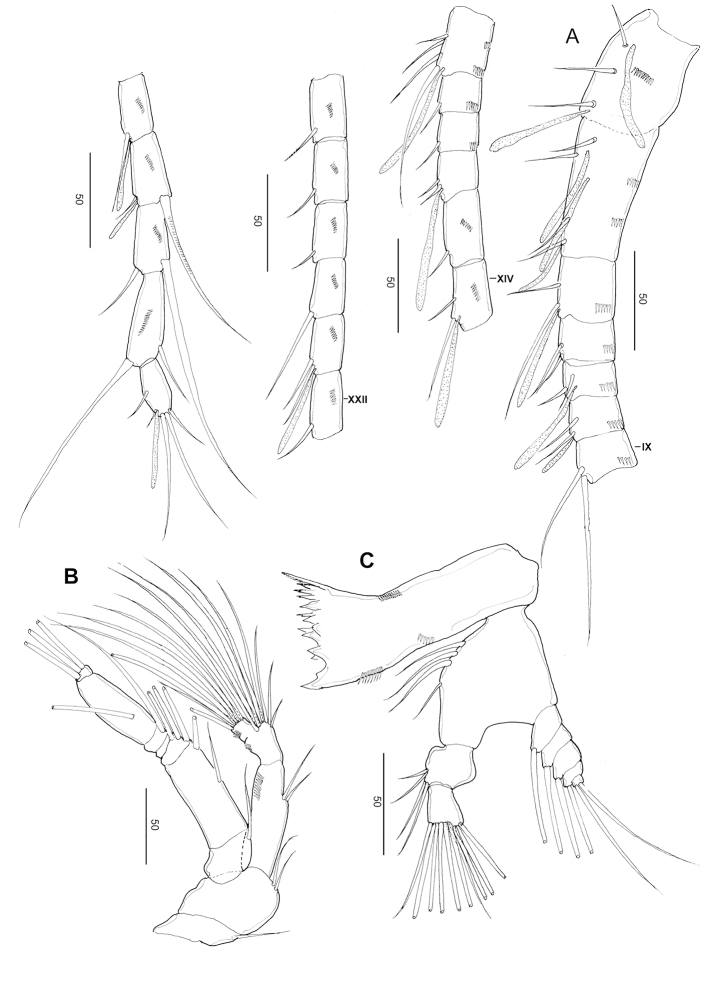
*Stephos concavus* sp. nov. Female holotype **A** antennule **B** antenna **C** mandible. Scale bars in µm.

***Antennule*** (Fig. 7A) symmetric, extending beyond distal area of genital double-somite; 24-segmented, apparently ancestral, segments I–II, III–IV, X–XI, and XXVII–XXVIII are fused. Segmentation and setation pattern as follows (ancestral segment number-setae+aesthetasc): I–II-3+2ae, III–IV-4+3ae, V-2+ae, VI-2, VII-2+ae, VIII2+ae, IX-2, X–XI-4+ae, XII-1, XIII-1, XIV-2+ae, XV-1, XVI-2+ae, XVII-1, XVIII-1, XIX-1, XX-1, XXI-2+ae, XXII-1, XXIII-1+ae, XXIV-1+1+ae, XXV-1+1, XXVI-1+1, XXVII–XXVIII-5+ae. Ancestral segments I–XIV and XVI–XXV with row of spinules on posterior surface.

***Antenna*** (Fig. 7B) biramous; coxa and basis separate, coxa with one and basis with two setae; endopod 2-segmented, proximal segment with two setae, compound distal segment bilobed with eight and seven plumose setae subterminally and terminally, respectively, outer margin ornamented with a small serrated process subdistally on medial margin; tiny spinule adjacent to serrated process; exopod 7-segmented, with intersegmental articulation between segments 2 and 3 not completely expressed, with setal formula of 1, 3, 1, 1, 1, 1, 3.

***Mandible*** (Fig. 7C): well-developed coxal gnathobase, with a straight row of moderately incised teeth and patched spinules on the anterior and posterior corners. Mandibular palp biramous; basis with four setae on inner margin. Exopod 5-segmented, with setal formula of 1, 1, 1, 1, 2; endopod 2-segmented, proximal with 4 setae and distal segments with 10 setae.

**Figure 8.**
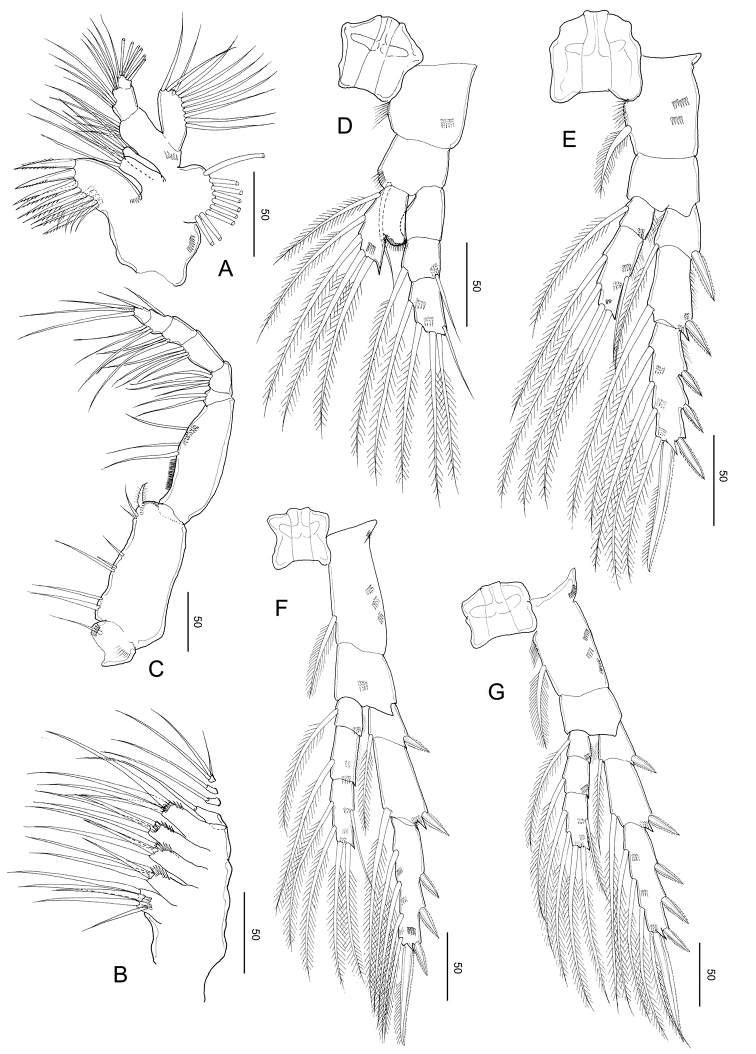
*Stephos concavus* sp. nov. Female holotype **A** maxillule **B** maxilla **C** maxilliped **D** leg 1 **E** leg 2 **F** leg 3 **G** leg 4. Scale bars in µm.

***Maxillule*** (Fig. 8A): praecoxal arthrite bearing nine stout marginal spines and four elements on posterior surface, rows of tiny spinules on the posterior surface. Coxal epipodite with nine setae; coxal endite with three stiff setae. Basis with cluster of denticles on the anterior surface; proximal basal endite with four setae; distal basal endite indistinct, with five setae; no trace of basal exite. Exopod with eleven marginal setae; with row of setules along the distal portion of the medial margin. Endopod not articulated to basis, indistinctly 3-segmented, setal formula 4, 4, 7.

***Maxilla*** (Fig. 8B): apparently 6-segmented, comprising coalesced praecoxa and coxa, allobasis, and 3-segmented endopod. Armature of praecoxal and coxal endites 5, 3, 3, 3, respectively. Basal endite with four setae, one stouter than the rest; endopodal endite with one seta on tip. Free endopod setal formula 1, 1, 3, respectively. Integument of praecoxa ornamented with a patch of spinules on the posterior margin. Praecoxal and coxal endites with a cluster of long spinules subdistally on the lateral surface; distal coxal endite with an additional row of spinules proximally on the medial surface.

***Maxilliped*** (Fig. 8C): syncoxa robust, with setal formula 1, 2, 2, 3 and an oblique row of tiny spinules on the anterior distal part; basis with three setae and patched setules on the mediolateral margin; endopod 6-segmented, with setal formula 2, 4, 4, 3, 3+1, 4.

***Legs 1–4*** (Fig. 8D–G) progressively larger towards the posterior, each comprising coxa, basis, and 3-segmented exopod; endopod of leg 1 (Fig. 8D) 1-segmented, that of leg 2 (Fig. 8E) 2-segmented; endopods of leg 3 (Fig. 8F) and P4 (Fig. 8G) 3-segmented. Armature formula of legs 1–4 as follows in *S. jejuensis* sp. nov.

Leg 1 (Fig. 8D) biramous, with long curved inner setae on the basis, and endopod with lobe on the outer margin, bearing a minute spinous process and a row of minute spinules on the dorsal surface.

Leg 2 (Fig. 8E) biramous, endopod 2-segmented; coxa and basis unarmed; second endopodal segments with a row of spinules on medial and distal edges, with pointed process on the distolateral corner; exopod 3-segmented, with a row of spinules on the medio to distal margins of the distal exopodal segment.

Legs 3 (Fig. 8F) and 4 (Fig. 8G) biramous, with 3-segmented rami: coxa and basis unarmed; second and distal endopodal segments with a row of spinules on the distal edges, with a pointed process on each distolateral corner; exopod with row of spinules on the medio to distal margins of the distal exopodal segment, except for a row of spinules on the posterior surface of leg 4 basis.

Leg 5 (Fig. 6F) symmetric, uniramous, 3-segmented with a proximal segment fused to intercoxal sclerite; basis separated, 2.53 times longer than wide (38 × 15 μm) and unarmed. Distal segment constricted slightly at ca. mid-length with five large spinules and a large seta medially and with two rows of teeth on both lateral each sides as figured.

**Male.** Not collected.

##### Remarks.

The new species *Stephos concavus* sp. nov. is easily recognized by its four diagnostic features in the female: the genital double-somite with a protruding lobe on the anterior to medial part of both lateral sides; the presence of seven large rows of spinules on the left side of the genital double-somite; the basis of leg 5 is separated, 2.53 times longer than wide; and the presence of large spinules mediodistally on distal segment of leg 5.

The new species closely resembles *S. cryptospinosus* (Zagami et al. 2000), but it differs in the following features in the female: the body length is 0.93 mm (vs. 0.86 mm in *S. cryptospinosus*); the presence of seven spinules on the left side of the genital double-somite (vs. absence in *S. cryptospinosus*); the antennule extends beyond the distal end of the genital double-somite (vs. beyond the posterior margin of the prosome in *S. cryptospinosus*); the presence of large spinules on the mediodistal margin of leg 5 distal segment (vs. absence in *S. cryptospinosus*); and the terminal segment with teeth on both sides and large spinules mediodistally on both fifth legs (vs. absence in *S. cryptospinosus*).

*Stephos concavus* differs from another congener *S. longipes* (Giesbrecht, 1902) in the following features of the female: the genital double-somite with protruding lobe on the anterior to medial part of both sides (vs. triangular lobe on the medial part of both sides in *S. longipes*); the presence of a row of spinules on the left side of the genital double-somite (vs. absence in *S. longipes*); the absence of a row of minute spinules on the dorsodistal surface of the genital double-somite (vs. presence in *S. longipes*); the leg 5 distal segment is tapering and stout (vs. tapering and not stout in *S. longipes*); and the teeth on the outer margin of both sides (vs. finely serrated fringe on the outer margin in *S. longipes*).

#### Stephos fortipes sp. nov.

Taxon classificationAnimaliaCalanoidaStephidae

886448FD-6016-57EA-B716-F8492C50BCBE

http://zoobank.org/C726634A-9A85-4966-B9B1-B63AA83DA929

Figures 9 , 10 , 11

##### Material examined.

***Holotype*** ♀ (NIBRIV0000293110) dissected on a glass slide collected by D. H. Cho, 9 May 2012.

##### Type locality.

Near the bottom (ca. 4 m depth), Wimi port, Jeju Island (approximately 33°16'13"N, 126°39'43"E), Korea.

##### Etymology.

The specific name *fortipes* is the combination of Latin words *fortis* (strong) and *pes* (leg), alluding to the strong feature of the female fifth leg.

**Figure 9.**
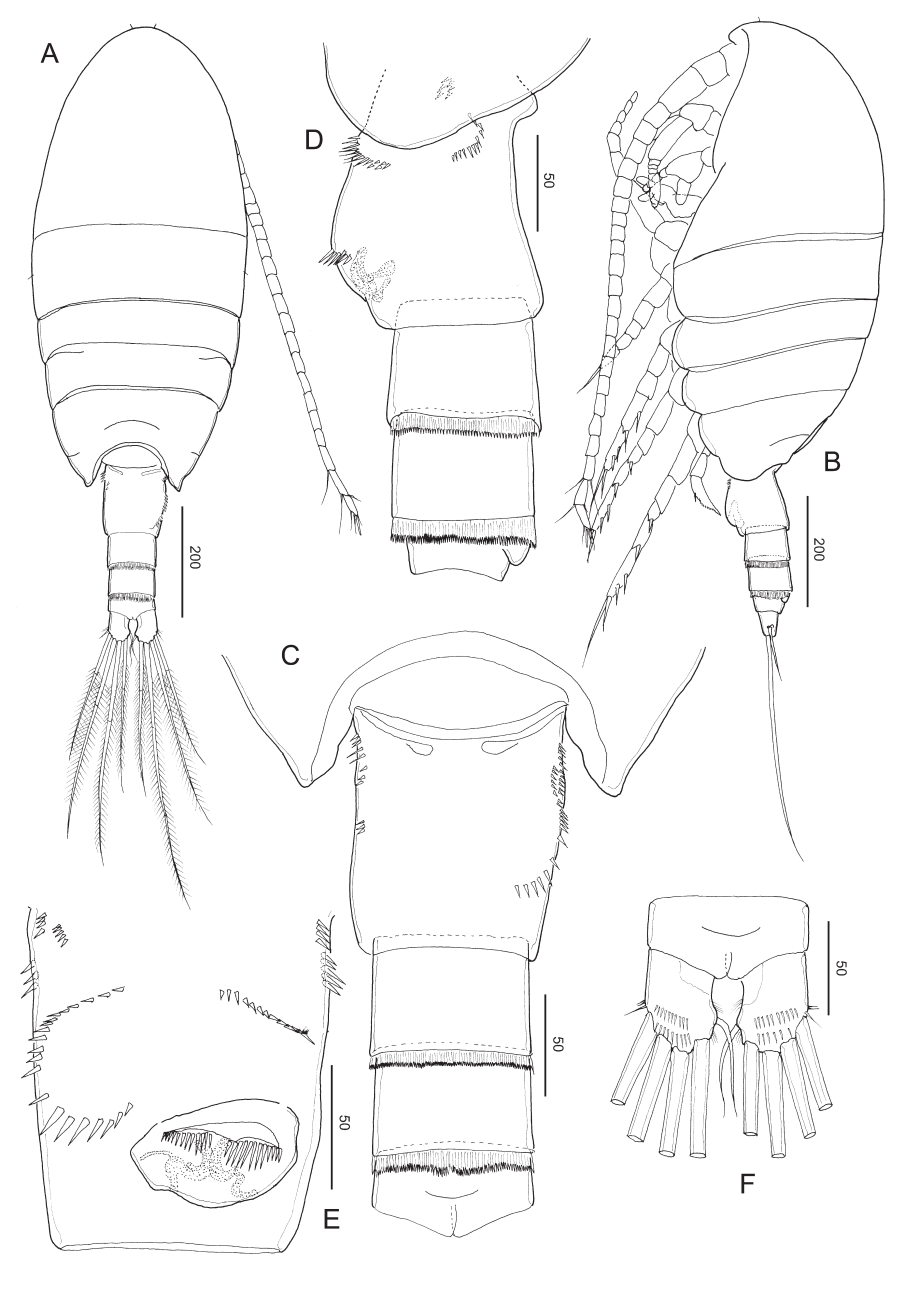
*Stephos fortipes* sp. nov. Female paratype **A** habitus, dorsal view **B** habitus, lateral view **C** urosome, dorsal view **D** urosome, lateral view **E** genital double-somite, ventral view **F c**audal rami, dorsal view. Scale bars in µm.

##### Description of female.

***Body*** (Fig. 9A, B) robust, length 1.12 mm. Prosome five-segmented; cephalosome and first pedigerous somites completely separated; fourth and fifth pedigerous somites incompletely fused (Fig. 9A), posterior corners of prosome slightly asymmetric. Rostrum represented by a rounded knob. Prosome-urosome ratio 2.45:1. Urosome 4-segmented, comprising a genital double-somite, two free abdominal somites, and anal somite; length ratio of genital double-somite, first free abdominal somite, second free abdominal somite, and anal somite as 39.1: 18.7: 17.1:15.1:10.0 = 100. Genital double-somite (Fig. 9C, E) slightly asymmetric with a differing groups of minute spinules on each side, anterior to mid-length; on the left side is a group of minute spinules that tend to be obscured by detritus and difficult to observe, patches and rows of fine spinules on the right side; genital double-somite not produced ventrally, operculum slightly round, with rows of spinules on the ventral surface. First and second abdominal somites (Fig. 9C), with transverse hyaline frill dorsally and ventrally. Anal somite shortest. Caudal rami (Fig. 9F), with six setae, symmetric, 1.19 times longer than wide (56 × 47 μm), with minute spinules on the dorsal surface; caudal setae II to VII present (seta I lacking); seta II spiniform, seta III ca. half the length of seta V, seta V longer (right longer than left) than seta IV, both plumose; dorsal seta VII short, plumose.

**Figure 10.**
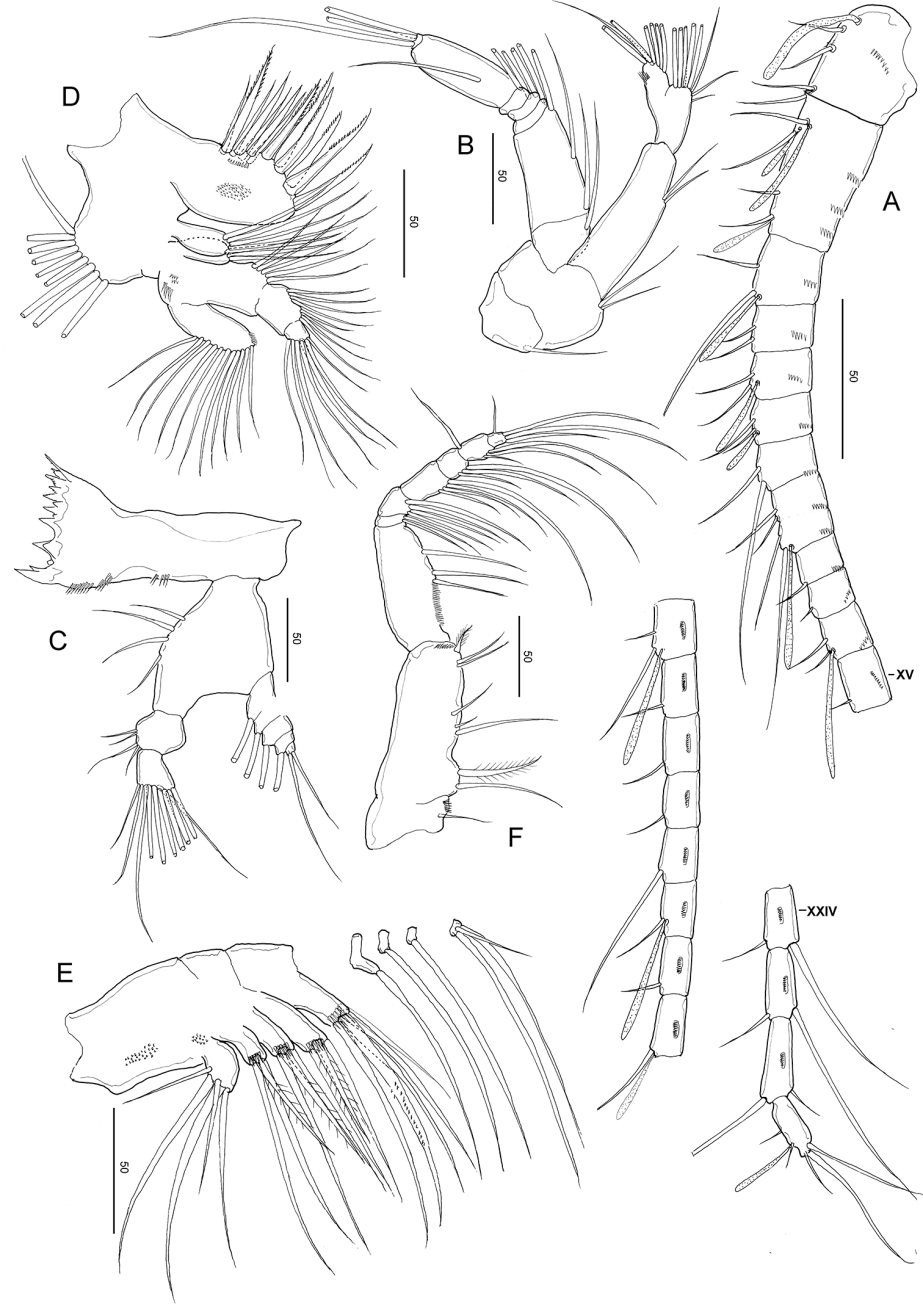
*Stephos fortipes* sp. nov. Female paratype **A** antennule **B** antenna **C** mandible **D** maxillule **E** maxilla **F** maxilliped. Scale bars in µm.

***Antennule*** (Fig. 10A) symmetric, extending near to distal area of genital double-somite; 24-segmented, apparently ancestral, segments I–II, III–IV, X–XI, and XXVII–XXVIII are fused. Segmentation and setation pattern as follows (ancestral segment number-setae+aesthetasc): I–II-3+ae, III–IV-4+3ae, V-2+ae, VI-2, VII-2+ae, VIII2+ae, IX-2, X-XI-4+ae, XII-1, XIII-1, XIV-2+ae, XV-1, XVI-2+ae, XVII-1, XVIII-1, XIX-1, XX-1, XXI-1+ae, XXII-1, XXIII-1, XXIV-1+1, XXV-1+1, XXVI-1+1, XXVII–XXVIII-5+ae. Ancestral segments I–XIV and XVI–XXV with a row of spinules on the posterior surface.

***Antenna*** (Fig. 10B) biramous; coxa and basis separate, coxa with one and basis with two setae; endopod 2-segmented, proximal segment with two setae, compound distal segment bilobed with eight and seven plumose setae subterminally and terminally, respectively, outer margin ornamented with a small serrated process subdistally on the medial margin; tiny spinule adjacent to the serrated process; exopod 7-segmented, with intersegmental articulation between segments 2 and 3 not completely expressed, with setal formula of 1, 3, 1, 1, 1, 1, 3.

***Mandible*** (Fig. 10C): well-developed coxal gnathobase, with a straight row of moderately incised teeth, ornamented with spinule rows on the medioventral part. Mandibular palp biramous; basis with four setae on inner margin. Exopod 5-segmented, with setal formula of 1, 1, 1, 1, 2; endopod 2-segmented, proximal with four setae and distal segments with ten setae.

***Maxillule*** (Fig. 10D): praecoxa and coxa incompletely fused; praecoxal arthrite with ten marginal spines plus four stiff setae on posterior surface, rows of tiny spinules on posterior surface. Coxal epipodite with nine setae; coxal endite with three stiff setae. Basis with cluster of denticles on the anterior surface; proximal basal endite with four setae; distal basal endite indistinct, with five setae; no trace of basal exite. Exopod with eleven marginal setae. A row of setules along the distal portion of the medial margin. Endopod not articulated to basis, indistinctly 3-segmented, setal formula 4, 4, 7.

***Maxilla*** (Fig. 10E): apparently 6-segmented, comprising coalesced praecoxa and coxa, allobasis, and 3-segmented endopod. Armature of praecoxal and coxal endites 5,3,3,3, respectively. Basal endite with four setae, one stouter than the rest; endopodal endite with one seta on tip. Free endopod setal formula 1, 1, 3, respectively. Integument of praecoxa ornamented with patch of spinules on the posterior margin. Praecoxal and coxal endites with a cluster of long spinules subdistally on the lateral surface; distal coxal endite with an additional row of spinules proximally on the medial surface.

***Maxilliped*** (Fig. 10F): syncoxa robust, with setal formula 1, 2, 2, 3 and an oblique row of tiny spinules on the posterior distal part; basis with three setae and a row of setules on the mediolateral margin; endopod six-segmented, with setal formula 2, 4, 4, 3, 3+1, 4.

**Figure 11.**
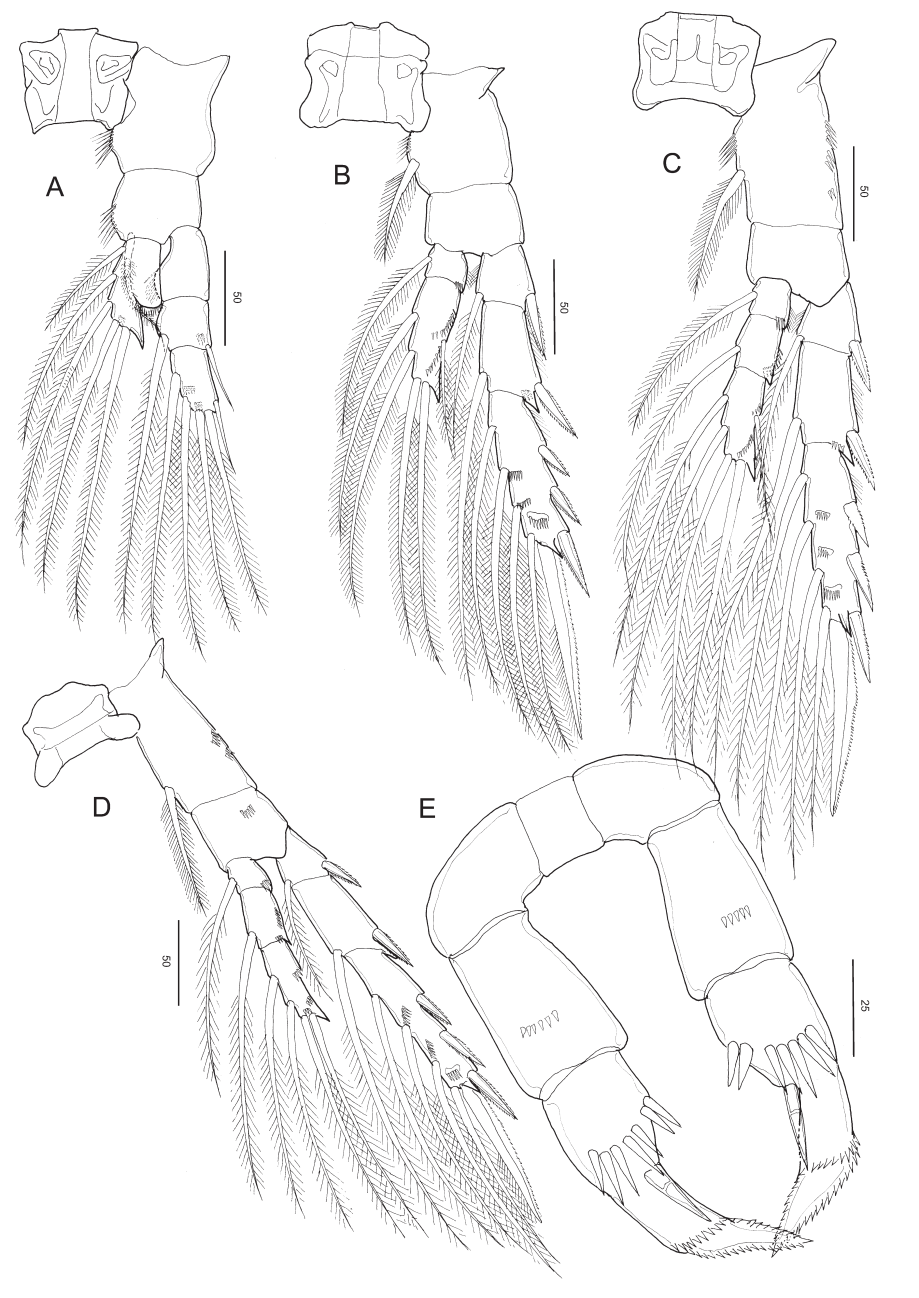
*Stephos fortipes* sp. nov. Female paratype **A** leg 1 **B** leg 2 **C** leg 3 **D** leg 4 **E** leg 5, dorsal view. Scale bars in µm.

***Legs 1–4*** (Fig. 11A–D), progressively larger towards posterior, each comprising coxa, basis, and 3-segmented exopod; endopod of leg 1 (Fig. 11A) 1-segmented, that of leg 2 (Fig. 11B) 2-segmented; endopods of leg 3 (Fig. 11C) and P4 (Fig. 11D) 3-segmented. Armature formula of legs 1–4 as in *S. jejuensis* sp. nov.

Leg 1 (Fig. 11A) biramous, coxa with hairs and spinules on the inner and posterior surfaces; basis with a row of spinules on the inner distal corner and long, curved inner setae, and endopod with a lobe on the outer margin, bearing a minute spinous process; second and distal exopodal segments with patched minute spinules; second and terminal exopodal segment with a row of spinules on the posterior margin.

Leg 2 (Fig. 11B) biramous, endopod 2-segmented; coxa with hairs on the inner margin, row of spinules on the posterior surface; basis unarmed; each first and second endopodal with row of spinules on the medial and distal edge, with pointed process on distolateral corner; exopod 3-segmented, with a row of spinules on the medio to distal margins of distal exopodal segment.

Legs 3 (Fig. 11C) and 4 (Fig. 11D) biramous, with 3-segmented rami: coxa with hairs on the inner margin and a row of spinules on the anterior surface; first to distal endopodal segments with a row of spinules on distal edges, with pointed process on each distolateral corner; exopod with a row of spinules on the medio to distal margins of distal exopodal segment.

Leg 5 (Fig. 11E) symmetric, uniramous, 3-segmented with proximal segment fused to intercoxal sclerite; basis separated from the single, tapering terminal segment. Second segment (basis) 1.38 times longer than wide (44 × 32 μm), with an anteromedial patch of minute spinules on the anterior surface. Distal segment constricted slightly at ca. mid-length with seven large spinules and inner stout spine and with two rows of denticles along the tapering portion

**Male.** Not collected.

##### Variations.

Within this new species, there was a minor variation in the number of spinules on the genital double-somite and on the surfaces of legs 1–4 in the female.

##### Remarks.

The new species closely resembles its congeners *S. angulatus* Bradford-Grieve, 1999, *S. hastatus*, and *S. pacificus* Ohtsuka & Hiromi, 1987; however, it differs in the following characteristics in the female: the antennule extends to the end of the genital double-somite (vs. first abdominal segment end in *S. angulatus*, and fifth pedigerous end in *S. hastatus* and *S. pacificus*); the operculum is slightly round (vs. triangular in three species); and the stout and present large row of spinules on the terminal tapering part of leg 5 (vs. not stout and absent in three species).

## Discussion

The benthopelagic copepod fauna of the Korean peninsula was previously surveyed (Soh et al. 2013; Moon et al. 2014, 2015). They recorded five species: *Sarsarietellus orientalis*, Soh et al., 2013, offshore of Yogji and Maemul Island, southern Korea; *Stephos geojinensis* from the Geojin fishery port in eastern Korea; *S. pacificus* from the shallow waters of Jangdeong beach in southern Korea; *S. projectus* from the Naro Island in southern Korea; and *Boholina ganghwaensis*, Moon & Soh, 2014, from Ganghwa Island in western Korea. The morphological characteristics of the genus *Stephos*, and a key to identifying the species were provided by Boxshall and Halsey (2004). Taxonomic analysis of closely related species of *Stephos* is based on the subtle morphological characters by Bradford-Grieve (1999) and Suárez-Morales et al. (2017). Bradford-Grieve (1999) categorized the species of the genus by analyzing the fourth segments of the male left fifth leg into four types. In this study, seven species in the Australian-Western Pacific region belong to “group IV”, where the fourth segment of the male left fifth leg is narrow, as follows: *S. angulatus* Bradford-Grieve, 1999; *S. geojinensis* Moon, Youn, & Venmathi Maran, 2015; *S. jejuensis* sp. nov., *S. morii* Greenwood, 1977; *S. pacificus* Ohtsuka & Hiromi, 1987; *S. pentacanthos* Chen & Zhang, 1965; and *S. tsuyazakiensis* Tanaka, 1967 (Table 1). The zoogeographic analysis presented based on the structural patterns of the female fifth leg by Suárez-Morales et al. (2017), grouped the 29 species of *Stephos* together. The three new species described here belong to “group A”, where lateral setae are present and segments are apically elongate. This primitive “group A” is the most widespread, present in the most diverse regions (Suárez-Morales et al. 2017).

Most species of *Stephos* are frequently found in hyperbenthic and epibenthic habitats of tropical to polar regions (Boxshall and Halsey 2004; Jaume et al. 2008; Kršinić 2015; Moon et al. 2015; Suárez-Morales et al. 2017), and are occasionally recorded in anchialine caves (Boxshall et al. 1990; Riera et al. 1991; Carola and Razouls 1996; Jaume et al. 2008; Suárez-Morales et al. 2017). However, in this study, the three new species were collected at night using a plankton net in shallow waters. Other stephids have also occurred in plankton samples collected at night in coastal waters (Kos 1972; Ohtsuka and Hiromi 1987; Costanzo et al. 2000; Zagami et al. 2000; Moon et al. 2015). These facts suggest that benthopelagic calanoids could undertake daily vertical migrations (Zagami et al. 2000; Moon et al. 2015) and also diel feeding rhythm, reproduction, molting, dispersal, and niche diversification (Alldredge and King 1980).

The stephids comparisons of morphological features between the three new species of Korean fauna and the all species of genus *Stephos* are based on both sexes in the World. *Stephos* shares many of the characteristics of *Miostephos* Bowman, 1976, but differs in that the right fifth leg in female is 4-segmented and the male right fifth leg ends in an unarmed claw and/or mitten-like segment in *Stephos* (Boxshall and Halsey 2004; Kršinić 2015). According to Suárez-Morales et al. (2017), the structure of the female fifth leg is of great significance in the taxonomy of stephids. These characteristics were used in the keys to species of *Stephos* by Suárez-Morales et al. (2017). Here the following combination of features are used in order to separate species: (1) the body length in both sexes; (2) the shape of postero-lateral corners in both sexes; (3) the shape and ornamentations of the genital double-somite in females; (4) the presence and/or absence of spinules on the caudal rami in both sexes; (5) the antennule extension in both sexes; and (6) the ornamentation and shape of fifth legs in both sexes.

The principal differences between the three new species and their congeners are summarized in Table 1. Although some features occasionally overlap within the all species considered herein, the characteristic combinations proposed are different for each species, showing them to be essential diagnostic elements. These morphological characteristics were very useful and important criteria for identifying each species of stephids.

**Table 1.** Comparison of morphological characteristics of *Stephos* spp.

**Character**	***Stephos angulatus* Bradford-Grieve, 1999**	***Stephos antarcticum* Wolfenden, 1908**	***Stephos arcticus* Sars, 1909**	***Stephos boettgerschnackae* Krsinic, 2012**	***Stephos canariensis* Boxshall, Stock & Sanchez, 1990**	***Stephos concavus* sp. nov.**	***Stephos cryptospinosus* Zagami, Campolimi & Costanzo, 2000**
**Female**							
Body length (mm)	0.82	1.85–2.0	1.20	0.89–0.93	0.64–0.69	0.93	0.86
Posterolateral corners	symmetrical	slightly asymmetrical	slightly asymmetrical	asymmetrical	asymmetrical	asymmetrical	symmetrical
Shape of genital double–somite	asymmetrical	symmetrical, lateral swelling on both side	asymmetrical, lateral swelling on left side	symmetrical	slightly asymmetrical	asymmetrical, convex on posterior corner	symmetrical
Operculum shape of genital double–somite	triangular	–	–	–	rounded	slightly rounded	slightly rounded
Ornamentation of genital double–somite	fringe of fine spinules on left side	long spines on both side	–	–	absent	left side with 7 row spinules	absent
Antennule extention	distal margin of first abdominal segment	distal margin of second abdominal segment	distal margin of fifth pedigerous somite	distal margin of caudal rami	distal margin of fifth pedigerous somite	distal margin of genital double–somite	distal margin of fifth pedigerous somite
Row of spinules on basis of fifth legs	absent	absent	absent	absent	present	absent	present
Shape of third segment of fifth legs	tapering	tapering	conical	tapering	tapering, short	tapering, short	tapering, short
Large spinules of third segment of fifth legs	absent	absent	absent	absent	present	present	absent
Outer seta on third segment of fifth legs	present	absent	present	present	present	present	present
Terminal distal part of the segment of fifth legs	curved, distal potion bordered by coarse teeth	curved, strongly serrate process apically	scarcely denticles on distal half of lateral margin	scattered denticles both side	scattered denticles both side	scattered denticles both side	finely serrated fringe on outer margin
**Male**							
Body length (mm)	0.703	1.75	1.05	0.82–0.9	0.59–0.64	–	0.78
Posterolateral corners	symmetrical	asymmetrical	–	asymmetrical	symmetrical	–	symmetrical
Shape of genital somite	asymmetrical, with genital aperture on the left	–	symmetrical	asymmetrical	symmetrical	–	symmetrical
Antennule extention	distal margin of fifth pedigerous end	second abdominal segment end	distal margin of fifth pedigerous end	end of caudal rami	about to posterior border of genital somite	–	approximately to posterior margin of genital somite
Type of left segment of P5	left segment 4 narrow	left segment 4 narrow, segment 5 bufurcate without leaf–like elements	left segment 4 swollen, segment 5 with leaf–like elements	left segment 4 swollen, segment 5 with leaf–like elements	left segment 4 swollen	–	left segment 4 swollen, segment 5 with leaf–like elements
**Character**	***Stephos deichmannae* Fleminger, 1957**	***Stephos exumensis* Fosshagen, 1970**	***Stephos fernandoi* Suárez-Morales, Gutiérrez-Aguirre, Cervantes-Martínez & Illife, 2017**	***Stephos fortipes* sp. nov.**	***Stephos fultoni* Scott T. & Scott A., 1898**	***Stephos geojinensis* Moon et al., 2015**	***Stephos grieveae* Kršinić, 2015**
**Female**							
Body length (mm)	0.62–0.73	0.73	0.47	1.12	1.00	0.88	0.60–0.67
Posterolateral corners	asymmetrical	asymmetrical	symmetrical	symmetrical	symmetrical	symmetrical	slightly asymmetrical
Shape of genital double–somite	asymmetrical	asymmetrical, protruding lobe on left side	symmetrical	asymmetrical	symmetrical	asymmetrical, protruding lobe on both side	symmetrical
Operculum shape of genital double–somite	–	–	rounded	rounded	–	rounded	rounded
Ornamentation of genital double–somite	present	present	4 slender spiniform elements on ventral surface	row of minute spinules on ventral surface	absent	row of spinules on ventral surface	absent
Antennule extention	–	–	posterior margin of preanal somite	distal margin of genital double–somite	distal half of genital double–somite	distal margin of second urosomite	distal margin of third urosomite
Row of spinules on basis of fifth legs	present	present	absent	present	absent	absent	absent
Shape of third segment of fifth legs	tapering	tapering	cylindrical	tapering, stout	asymmetrical, right leg longer than left	tapering, elongated	tapering, elongated
Large spinules of third segment of fifth legs	absent	present	absent	present	absent	absent	absent
Outer seta on third segment of fifth legs	absent	absent	absent	present (spine)	absent	present	present
Terminal distal part of the segment of fifth legs	row of spinules on distal half of lateral margin	finely serrated fringe on outer margin	spiniform bipinated apical process	distal potion bordered by fringe teeth	broad knife–like shape of right leg	coarsely serrated spine incorporated distally	single small spine on anterior surface
**Male**							
Body length (mm)	0.61–0.66	–	0.49	–	1	0.819	0.55–0.62
Posterolateral corners	asymmetrical	–	symmetrical	–	symmetrical	symmetrical	symmetrical
Shape of genital somite	asymmetrical	–	symmetrical	–	symmetrical	asymmetrical, with lateral lone at each side	slightly asymmetrical
Antennule extention	–	–	beyond distal margin of second urosomite	–	–	distal margin of second urosomite	distal margin of third urosomite
Type of left segment of P5	left segment 4 swollen	–	left segment 4 narrow, segment 5 bufurcate without leaf–like elements	–	left segment 4 swollen	left segment 4 narrow	left segment 4 narrow
**Character**	***Stephos gyrans* (Giesbrecht, 1893)**	***Stephos hastatus* Bradford-Grieve, 1999**	***Stephos jejuensis* sp. nov.**	***Stephos kurilensis* Kos, 1972**	***Stephos lamellatus* Sars G.O., 1902**	***Stephos longipes* Giesbrecht, 1902**	***Stephos lucayensis* Fosshagen, 1970**
**Female**							
Body length (mm)	0.9–1.0	1.06	0.92	1.32	1.00	0.75–0.80	0.63–0.71
Posterolateral corners	symmetrical	asymmetrical	asymmetrical	asymmetrical	asymmetrical	–	asymmetrical
Shape of genital double–somite	asymmetrical	asymmetrical	asymmetrical, protruding lobe	asymmetrical, swollen on the left side	asymmetrical	symmetrical, triangular swollen on both sides	Asymmetrical, slightly more swollen on the left than on the right side
Operculum shape of genital double–somite	–	triangular	bumpy	–	–	–	–
Ornamentation of genital double–somite	row of spinules anteriorly on ventral surface	weak spine on right anterior margin	–	absent	absent	present	present
Row of spinules/setules on the dorsal surface of caudal rami	absent	distal margin of fifth pedigerous somite	distal margin of genital double–somite	genital double–somite proximally	–	absent	–
Antennule extention	–	present	present	absent	distal margin of caudal rami	–	–
Row of spinules on basis of fifth legs	absent	tapering	tapering	tapering	absent	absent	present
Shape of third segment of fifth legs	tapering, curved	absent	absent	present	tapering	tapering, curved	tapering
Large spinules of third segment of fifth legs	present	present	absent	present	present	absent	present
Outer seta on third segment of fifth legs	absent	striated hyaline	row of spinules across near the middle part	strongly serrate process apically	present	present	absent
Terminal distal part of the segment of fifth legs	strongly serrate process apically				strongly serrate process apically	strongly serrate process apically	strongly serrate process apically
**Male**		0.943	0.93	1.24			
Body length (mm)	–	symmetrical	asymmetrical	asymmetrical	1.0mm	0.65–0.70	0.58–0.68
Posterolateral corners	symmetrical	asymmetrical, with genital aperture on the left	asymmetrical, with protruding lobe on the left side	slightly asymmetrical	symmetrical	symmetrical	slightly asymmetrical
Shape of genital somite	–	distal margin of fifth pedigerous end	distal margin of genital double–somite	distal margin of fifth pedigerous end	symmetrical	asymmetrical	asymmetrical
Antennule extention	distal margin of second urosomite	left segment 4 swollen	left segment 4 narrow	left segment 4 swollen	distal margin of caudal rami	–	–
Type of left segment of P5	left leg 4 swollen, segment 5 with leaf–like elements	leg segment 4 swollen	left leg segment 4 narrow	left segmeht 4 swollen	left segment 4 swollen	left segment 4 narrow, segment 5 bifurcate without lelf–like elements	left segment 4 swollen
**Character**	***Stephos maculosus* Andronov, 1974**	***Stephos margalefi* Riera, Vives & Gili, 1991**	***Stephos marsalensis* Costanzo, Campolmi & Zagami, 2000**	***Stephos minor* Scott T., 1892**	***Stephos morii* Greenwood, 1978**	***Stephos pacificus* Ohtsuka and Hiromi, 1987**	***Stephos pentacanthos* Chen and Zhang, 1965**
**Female**							
Body length (mm)	0.86	0.77–0.80	0.76	0.73	–	0.73	–
Posterolateral corners	slightly asymmetrical	symmetrical	Slightly symmetrical	slightly asymmetrical	–	symmetrical	–
Shape of genital double–somite	symmetrical	symmetrical	symmetrical	symmetrical	–	symmetrical	–
Operculum shape of genital double–somite	–	–	–	–	–	–	–
Ornamentation of genital double–somite	absent	–	Row of minute spinules dorsolaterally	–	–	row of minute spinules on both side	–
Antennule extention	distal margin of caudal rami	distal margin of second urosomite	Posterior margin of first urosomite	beyond of genital double– somite	–	fifth pedigerous end	–
Row of spinules on basis of fifth legs	absent	absent	absent	absent	–	present	–
Shape of third segment of fifth legs	tapering, curved	tapering, stouted	asymmetrical	tapering, stouted	–	tapering	–
Large spinules of third segment of fifth legs	absent	absent	present	absent	–	absent	–
Outer seta on third segment of fifth legs	absent	present	absent	present	–	absent	–
Terminal distal part of the segment of fifth legs	strongly serrate process apically	strongly serrate process apically	right segment sickle–shaped with patch of spinules on proximal anterior surface	scarcely denticuls on distal half of lateral margin	–	striated hyaline	–
**Male**							
Body length (mm)	0.54	0.74	0.73	0.6	0.85	0.62	0.75
Posterolateral corners	slightly asymmetrical	asymmetrical	symmetrical	symmetrical	asymmetrical	slightly asymmetrical	asymmetrical, produced
Shape of genital somite	symmetrical	asymmetrical	symmetrical	symmetrical	asymmetrical, more rounded on left side than right	asymmetrical with protruding lone on left side	–
Antennule extention	distal margin of fourth urosimite	–	Posterior margin of second urosomite	end of last pedigerous somite	distal margin of third urosomite	distal margin of fifth pedigerous somite	distal margin of second urosomite
Type of left segment of P5	left leg segment 4 swollen	left segment 4 swollen	left segment 4 narrow	left segment 4 swollen	left segment 4 narrow	left segment 4 narrow	left segment 4 narrow
**Character**	***Stephos projectus* Moon et al., 2015**	***Stephos robustus* Ohtsuka and Hiromi, 1987**	***Stephos rustadi* Strömgren, 1969**	***Stephos scotti* Sars, 1902**	***Stephos tropicus* Mori, 1942**	***Stephos tsuyazakiensis* Tanaka, 1967**	***Stephos vivesi* Jaume, Boxshall & Gràcia, 2008**
**Female**							
Body length (mm)	1.51	1.01	0.68	0.95	0.82	0.78	0.45
Posterolateral corners	asymmetrical	asymmetrical	symmetrical	symmetrical	symmetrical	asymmetrical	slightly asymmetrical
Shape of genital double–somite	asymmetrical, elongated	asymmetrical, lateral swelling on both sides	asymmetrical, lateral swelling on both sidess	symmetrical	–	swellon on the left side	symmetrical
Operculum shape of genital double–somite	bumpy	fringed	–	–	–	–	paired genital opercular plates
Ornamentation of genital double–somite	patch spinules on dorsal surface	row of spinules on anterior margin	–	absent	–	left side with minute spinules	row of spinules on ventrolateral and dorsolateral margin
Antennule extention	distal margin of fifth pedigerous somite	distal margin of fifth pedigerous	beyond of fifth pedigerousn somite	distal margin of second urosomite	–	distal margin of genital segment	beyond posterior margin of fifth pedigerous somite
Row of spinules on basis of fifth legs	absent	present	absent	absent	absent	–	absent
Shape of third segment of fifth legs	tapering, elongated	tapering	absent	tapering	tapering	tapering	tapering, stout
Large spinules of third segment of fifth legs	absent	present	absent	absent	absent	present	absent
Outer seta on third segment of fifth legs	present	present	present	present	absent	absent	present
Terminal tapering part of the segment of fifth legs	curved, distal potion furnished	curved, distal potion both sides	finely serrated fringe on outer margin	row of denticles on distal margin	finely serrated fringe on outer margin	row of spinules on distal margin	two rows of denticles on distal part
**Male**							
Body length (mm)	0.93	0.91	0.62	0.85	0.73	0.73	0.44–0.45
Posterolateral corners	asymmetrical, with lateral lobe on left margin	asymmetrical	symmetrical	–	–	asymmetrical, slightly produced on the left side	slightly asymmetrical
Shape of genital double–somite	asymmetrical with protruding lone on left side	asymmetrical, spinules on lateral margin	symmetrical	–	–	asymmetrical, produced on the left side	asymmetrical, produced laterally on left side
Antennule extention	distal margin of fifth pedigerous somite	distal margin of fifth pedigerous somite	distal margin of third urosomite	distal margin of third urosomite	distal margin of second urosomite	distal margin of second urosomite	beyond posterior margin of fifth pedigerous somite
Type of left segment of fifth leg	left segment 4 swollen	left segment 4 swollen	left segment 4 narrow	left segment 4 swollen	left segment 4 swollen	left segment 4 narrow	left segment 4 narrow

To date, the genus *Stephos* consists of 35 valid species, including those described herein (Boxshall and Halsey 2004; Jaume et al. 2008; Kršinić 2012, 2015; Moon et al. 2015; Suárez-Morales et al. 2017; this study). Additionally, most of the genus *Stephos* species were not described following modern standards, and most of them need to be redescribed. Thus, the taxonomy, morphological variability, and distribution of stephids are well understood (Bradford-Grieve 1999; Moon et al. 2015; Suárez-Morales et al. 2017). These facts suggest that more detailed research on its taxonomy, biodiversity, and molecular features is necessary for a better understanding of its evolutionary history.

## Supplementary Material

XML Treatment for Stephos jejuensis

XML Treatment for Stephos concavus

XML Treatment for Stephos fortipes
